# Small antisense oligonucleotides against G-quadruplexes: specific mRNA translational switches

**DOI:** 10.1093/nar/gku1311

**Published:** 2014-12-15

**Authors:** Samuel G. Rouleau, Jean-Denis Beaudoin, Martin Bisaillon, Jean-Pierre Perreault

**Affiliations:** RNA Group/Groupe ARN, Département de biochimie, Faculté de médecine et des sciences de la santé, Pavillon de recherche appliquée sur le cancer, Université de Sherbrooke, Québec, J1E 4K8, Canada

## Abstract

G-quadruplexes (G4) are intricate RNA structures found throughout the transcriptome. Because they are associated with a variety of biological cellular mechanisms, these fascinating structural motifs are seen as potential therapeutic targets against many diseases. While screening of chemical compounds specific to G4 motifs has yielded interesting results, no single compound successfully discriminates between G4 motifs based on nucleotide sequences alone. This level of specificity is best attained using antisense oligonucleotides (ASO). Indeed, oligonucleotide-based strategies are already used to modulate DNA G4 folding *in vitro*. Here, we report that, in human cells, the use of short ASO to promote and inhibit RNA G4 folding affects the translation of specific mRNAs, including one from the 5′UTR of the H2AFY gene, a histone variant associated with cellular differentiation and cancer. These results suggest that the relatively high specificity of ASO-based strategies holds significant potential for applications aimed at modulating G4-motif folding.

## INTRODUCTION

G-quadruplexes (G4) are non-canonical, stable structures adopted by guanine-rich sequences (1). G4 folding requires a monovalent cation, preferably potassium (K^+^) (2). A typical G4 consists of four tracts, of at least two guanines per tract, interspaced by loops of 1–7 nucleotides (nt). The human genome harbors hundreds of thousands of potential G4-forming sequences (3,4). Telomeres and promoters are enriched in DNA G4 and these have been studied for decades (5). Recently, quantitative visualization in human cells, using structure-specific antibodies, has provided compelling physiological evidence of the formation of both DNA and RNA G4 *in vivo* (6,7). Compared to their DNA counterparts, RNA G4 tend to be more stable and display less topological diversity. Importantly, folding of RNA G4 is not precluded by the presence of a complementary strand. This special property increases the likelihood of biological activity *in vivo*. Several biological functions for RNA G4 have indeed been reported recently (8) including translational repression (9,10), RNA splicing (11,12), mRNA polyadenylation (13,14) and mRNA localization (15).

G4 are viewed as potential targets for the treatment of disease, including cancer (16). Screening of chemical compounds for the specific ability to recognize and bind G4 has yielded several ligands exhibiting a high selectivity toward G4 structures (17,18). However, given the high number of potential G4 in a cell (3,4), off-target effects pose a major obstacle. The fact that most chemical ligands do not successfully discriminate between DNA and RNA G4 only worsens the problem, though some advances to circumvent this hurdle have recently been reported (19). Alternatively, targeting of G4 using small antisense oligonucleotides (ASO) (20,21) should, in theory, provide sequence specificity via Watson–Crick base-pairing. However, chemically modified analogs and/or nucleic acid mimics have proven necessary for effective DNA G4 targeting to occur *in vitro* (reviewed in (22)). These reports were nevertheless limited to DNA G4 and *in vitro* experiments. In other experiments, PNAs oligonucleotides were used *in vitro* to form hetero-G4 with DNA as well as RNA (23,24). The use of small oligonucleotides that form intermolecular G4 with cellular (25,26) as well as viral mRNAs (27) has also been reported. On the contrary, G4 folding in a viral mRNA was recently shown to be inhibited by either RNA or DNA ASO, promoting translation in an *in vitro* assay (28). Here, our goal was to target RNA G4 in human cells for the purpose of sequence-specific modulation of translation i.e. to promote, as well as to inhibit, RNA G4 folding in order to modulate translation. Artificial as well as naturally occurring RNA G4 sequences were successfully targeted. Among the naturally occurring sequences was a G4 from the 5′ untranslated region (UTR) of the H2AFY gene (also known as macroH2A1), encoding a histone variant involved in cellular differentiation (29) and several types of cancer (30–33).

## MATERIALS AND METHODS

### RNA synthesis

All RNA molecules used for in-line and circular dichroism (CD) experiments were synthesized by *in vitro* transcription, using T7 RNA polymerase as described previously (34). Briefly, two overlapping oligonucleotides (2 μM each) were annealed and double-stranded DNA was then obtained by filling in the gaps using purified *Pfu* DNA polymerase in the presence of 5% dimethyl sulfoxide (DMSO). Double-stranded DNA was then ethanol-precipitated. Resulting DNA templates contained the T7 RNA promoter sequence upstream of the G4 sequence. After dilution of the polymerase chain reaction (PCR) product in water, transcription was carried out in the presence of purified T7 RNA polymerase (10 μg), RNase Out (24 U, Invitrogen), pyrophosphatase (0.01 U, Roche Diagnostics) and PCR product (2–5 mM) in a buffer containing 80-mM HEPES-KOH, pH 7.5, 24-mM MgCl_2_, 2-mM spermidine, 40-mM DTT and 5 mM of each NTP, in a final volume of 100 μl at 37°C for 2 h. Reaction mixtures were then treated with DNase RQ1 (Promega) at 37°C for 15 min, and the RNA purified by phenol/chloroform extraction and ethanol precipitation. Resulting pellets were dissolved in a 1:2 mixture of water and loading buffer (95% formamide, 10-mM ethylenediaminetetraacetic acid (EDTA), pH 8.0, 0.025% bromophenol blue). Samples were fractionated through 8% denaturing polyacrylamide gels (PAGE) 19:1 acrylamide:bisacrylamide, in buffer containing 45-mM Tris-borate, pH 7.5, 8-M urea and 2-mM EDTA. Reaction products were visualized by ultraviolet (UV) shadowing. Bands corresponding to correct sizes were excised, and transcripts eluted overnight in elution buffer (500-mM ammonium acetate, pH 7,2, 10-mM EDTA and 0.1% sodium dodecyl sulphate (SDS)). Transcripts were ethanol precipitated, dried and resuspended in water. RNA was quantified by absorbance upon exposure to UV light at 260 nm. The 2’O-Me ASO were purchased from Integrated DNA Technology and the locked nucleic acid (LNA)/DNA ASO was purchased from Exiqon. The hybrid ASO beard an entire phosphothiorate backbone and 8 LNA residues. The LNA pattern is shown in Supplementary Table S1.

### RNA labeling

Purified transcripts (50 pmol) were dephosphorylated in a solution containing 1 U of Antarctic phosphatase (New England BioLabs), 50-mM Bis-Propane (pH 6.0), 1-mM MgCl_2_, 0.1-mM ZnCl_2_ and RNase OUT (20 U, Invitrogen) in a final volume of 10 μl. Phosphatase was inactivated by incubation for 5 min at 65°C. Dephosphorylated transcripts (5 pmol) were 5′-end-radiolabeled using 3 U of T4 polynucleotide kinase (Promega) for 1 h at 37°C in the presence of 3.2 pmol [α-^32^P]ATP (6000 Ci/mmol; New England Nuclear) in a final volume of 10 μl. Reactions were stopped by adding 20-μl loading buffer (95% formamide, 10-mM EDTA and 0.025% bromophenol blue), and RNA molecules purified by 10% PAGE. Bands of the correct sizes containing the 5′-end-labeled RNAs were excised and recovered as described above except for detection which was performed by autoradiography.

### In-line probing

Trace amounts of labeled RNA (50 000 cpm) were heated at 70°C for 5 min and then slow-cooled to room temperature over 1 h in buffer containing 50-mM Tris–HCl (pH 7.5), 100-mM LiCl or KCl and 0, 1 or 10 μM of the corresponding ASO in a final volume of 10 μl. Following incubation, the final volume of each sample was adjusted to 20 μl such that final concentrations were 50-mM Tris–HCl (pH 7.5), 20-mM MgCl_2_, 100-mM LiCl or KCl and 0, 0.5 or 5 μM of the ASO. Reactions were then incubated for 40 h at room temperature, ethanol-precipitated and RNA pellets dissolved in formamide-dye loading buffer (95% formamide, 10-mM EDTA and 0.025% bromophenol blue). For alkaline hydrolysis, 5′-end-labeled RNAs (50 000 cpm) were dissolved in 5 μl of water, 1 μl of 1-N NaOH was added and reactions were incubated for 1 min at room temperature prior to being quenched by addition of 3 μl of 1-M Tris–HCl (pH 7.5). RNA molecules were then ethanol-precipitated and dissolved in formamide-dye loading buffer. RNase T1 ladders were prepared using 50 000 cpm of 5′-end-labeled RNA dissolved in 10 μl of buffer containing 20-mM Tris–HCl (pH 7.5), 10-mM MgCl_2_ and 100-mM LiCl. The mixtures were incubated for 2 min at 37°C in the presence of 0.6 U of RNase T1 (Roche Diagnostic), and then quenched by the addition of 20 μl of formamide-dye loading buffer. Radioactivity of in-line probing samples and ladders were measured, and equal amounts in terms of cpm were fractionated on denaturing (8-M urea) 10% PAGE. Semi-Automated Footprinting Analysis (SAFA) software (35) was used to quantify each band. KCl band intensity was divided by that of the corresponding LiCl band. Histograms show the mean results of two independent experiments.

### Circular dichroism

All CD experiments were performed using 4 μM of the relevant RNA sample dissolved in 50-mM Tris–HCl (pH 7.5), in the absence of any monovalent salt, as well as in the presence of either 100-mM LiCl or KCl. Prior to CD measurement, each sample was heated to 70°C for 5 min and then slow-cooled to room temperature over a 1-h period. CD spectroscopy experiments were performed with a Jasco J-810 spectropolarimeter equipped with a Jasco Peltier temperature controller, in a 1-ml quartz cell with a path length of 1 mm. CD scans, ranging from 220 to 320 nm, were recorded at 25°C at 50 nm min^−1^ with a 2-s response time, 0.1-nm pitch and 1-nm bandwidth. The means of at least three wavelength scans were compiled. Subtraction of the buffer was not required as control experiments performed in the absence of RNA showed negligible curves. CD melting curves were obtained by heating samples from 25°C to 90°C at a controlled rate of 1°C min^−1^ and monitoring a 264-nm CD peak every 0.2 min. Melting temperature (Tm) values were calculated using ‘fraction folded’ (θ) versus temperature plots (36).

### DNA constructs

All 5′UTR sequences and their G/A-mutant counterparts were cloned 10 nucleotides (nts) upstream of the renilla luciferase reporter gene in the psiCHECK-2 vector (Promega) using the Nhe I restriction enzyme site. For ArtG4 and DsG4, two overlapping oligonucleotides with overhangs compatible with a Nhe I-digested vector were annealed and then cloned. For H2AFY, the 5′UTR sequence (NCBI; NM_001040158) was constructed using several overlapping primers and multiple PCR steps. For Akirin 2, the 5′UTR from a plasmid containing the whole Akirin 2 gene (PlasmID; DF/HCC DNA Resource Core) was first amplified by PCR with primers that added Nhe I sites onto both ends, subsequently digested and then cloned. All construct sequences were confirmed by DNA sequencing. All oligonucleotides used are shown in Supplementary Table S1.

### Cell culture and dual-luciferase assays

Human embryonic kidney HEK293 cells were cultured in T-75 ﬂasks (Sarstedt) in Dulbecco's modified Eagle's medium (DMEM) supplemented with 10% fetal bovine serum (FBS) and 1-mM sodium pyruvate (all purchased from Wisent), at 37°C in a 5% CO_2_ atmosphere-humidified incubator. Twenty-four hours before transfection, cells (1.2 × 10^5^) were seeded in 24-well plates. Transfections were carried out in two steps. On the first day, specific ASO (750 ng/well) were transfected using 2-μl Lipofectamine 2000 (Invitrogen) per well, according to manufacturer's protocol. Twenty-four hours later, specific psiCHECK-2 constructs (25 ng/well + 475 ng/well of pUC19 carrier DNA) were transfected using 0.5-μl Lipofectamine 2000 (Invitrogen) per well, according to manufacturer's protocol. After a further 24 h, cells were lysed and renilla (Rluc) and firefly luciferase (Fluc) activities measured using the Dual-luciferase Reporter Assay kit and a GloMax® 20/20 Luminometer (Promega), according to manufacturer's protocol. For each lysate, the Rluc value was divided by that of Fluc. Ratios were compared between every wild-type candidate and its G/A-mutant counterpart.

### Quantitative PCR analyses

RNA was extracted from whole cells dissolved in Qiazol (Qiagen), according to manufacturer's protocol. After phenol/chloroform extraction and isopropanol precipitation, total RNA was dissolved in water and treated with DNAseQ1 (Promega) for 1 h at 37°C. DNAse was then removed by phenol/chloroform extraction and EtOH precipitation. RNA quality and levels of contaminant genomic DNA were verified as previously described (37). RNA integrity was assessed with an Agilent 2100 Bioanalyzer (Agilent Technologies). Reverse transcription was performed on ∼0.2-μg total RNA, using Transcriptor reverse transcriptase, specific primer 5′-GTACGACTCACTATAGGGATTTTTTTTTTTTTTTTTV-3′ (V stands for A, G or C) for luciferase mRNA or random hexamers for endogenous mRNAs, dNTPs (Roche Diagnostics) and 10 units of RNAseOUT (Invitrogen) according to manufacturer's protocol in a total volume of 20 μl. All forward and reverse primers were individually resuspended to 20–100-μM stock solutions in Tris-EDTA buffer (IDT) and then diluted as a primer pair to 1 μM in RNase DNase-free water (IDT). Quantitative PCR (qPCR) reactions were performed in 10-μl total volume, in 96-well plates on a CFX-96 thermocycler (BioRad), containing 5-μl 2X iTaq Universal SYBR Green Supermix (BioRad), 10-ng (3 μl) cDNA and 200-nM final (2 μl) primer pair solution. Cycling conditions were as follows: 3 min at 95°C; 50 cycles: 15 s at 95°C, 30 s at 60°C, 30 s at 72°C. Relative expression levels were calculated using the qBASE framework (38) and either Fluc mRNA for the Rluc gene, or housekeeping genes MRPL19, SDHA and YWHAZ for the endogenous H2AFY. Primer design and validation were evaluated as described elsewhere (37). Every qPCR run included no-template primer-pair control samples and these were consistently negative. All primer sequences are available in Supplementary Table S2. qPCR assays were performed at the Université de Sherbrooke Functional Genomics Laboratory.

### Histone extraction and western blotting

Caco2 cells were cultured in 100-mm Petri dishes (Sarstedt) in DMEM supplemented with 10% FBS and 1-mM sodium pyruvate (all purchased from Wisent), at 37°C in a 5% CO_2_ atmosphere-humidiﬁed incubator. Transfections were carried out as cells were plated out in 6-well plates (Sarstedt). Approximately ∼1.2 × 10^6^ cells per well were plated and concomitantly transfected with 20-nM ASO and 4.5-μl Lipofectamine 3000 (Life technologies). After 24 h, cells were washed and scraped in ice-cold PBS. One fifth of the cells was used for RNA extraction (see the Quantitative PCR analyses section) and the rest for histone extraction with the EpiQuik™ Total Histone Extraction HTKit (Epigentek) according to manufacturer's protocol. Approximately 1 μg of total histones was fractionated through 12% acrylamide SDS-PAGE. Macroh2a1 polyclonal rabbit antibody (Active motif no39594) diluted 1000X was used for western blot analysis. Histone 3 monoclonal antibody (Active motif no61476) diluted 1000X was used as a loading control. Results are from three independent experiments, each performed in triplicate.

### Statistical analyses

Statistical analyses were performed for the dual-luciferase assays and qPCR analyses. Briefly, for each construct, the mean value and standard deviation were calculated from at least three independent experiments, each performed in triplicate. *P*-values were computed using a two-sided Student's *t*-test and GraphPad Prism 6 software.

## RESULTS

### Inhibiting the folding of artificial RNA G4

**Figure 1.**
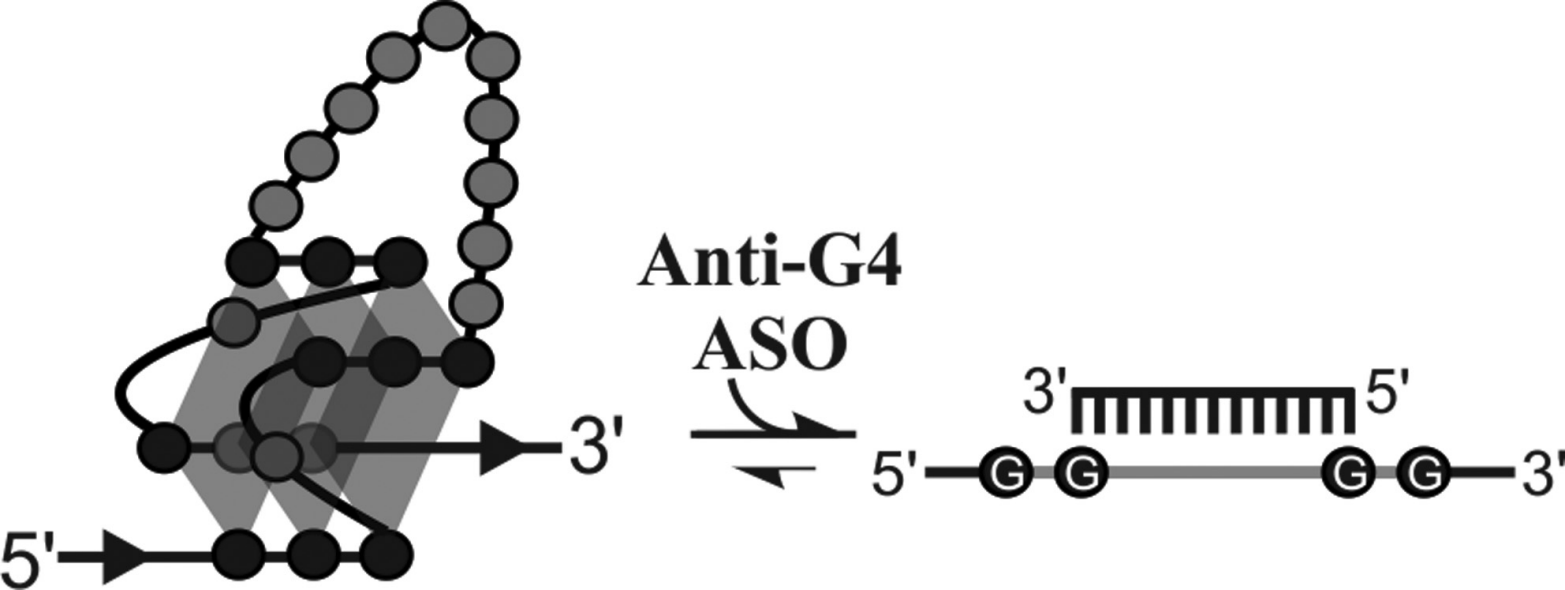
Schematic representation of the oligonucleotide-based strategy to inhibit G4-structure formation. G-tract residues (dark circles) , G tracts (white G in circles), loop nucleotides (light circles and light lines) and the anti-G4 oligonucleotide are shown.

We hypothesized that inhibiting the folding of a G4 structure should be possible using an ASO that simultaneously binds to one of the target G4's loops and to both of that loop's adjacent G tracts (Figure 1). Accordingly, the longer the binding region, the better the resulting inhibition should be. Overly lengthy ASO, however, expectedly produce non-specific effects via imperfect binding to non-target sequences. ASO length should thus be engineered to promote adequate affinity binding to a single target site. Loops are typically 1- to 7-nt long and, therefore, of insufficient length to provide both adequate affinity and specificity. However, folding has been shown to occur for a DNA G4 structure with a longer, central loop 2 sitting between a pair of single nucleotide-long loops. Extending loop 2 length up to 30 nt did not preclude G4 folding (39). Another group showed that RNA G4 with three loops as long as 15 nt each do fold *in vitro* (40). Recently, our group reported over a thousand RNA G4, each harboring a single, long central loop found in the 5′UTR of several human genes (41). A number of these sequences were shown to fold into G4 *in vitro* and to impair the expression of a luciferase reporter gene *in cellulo* (41).

**Figure 2.**
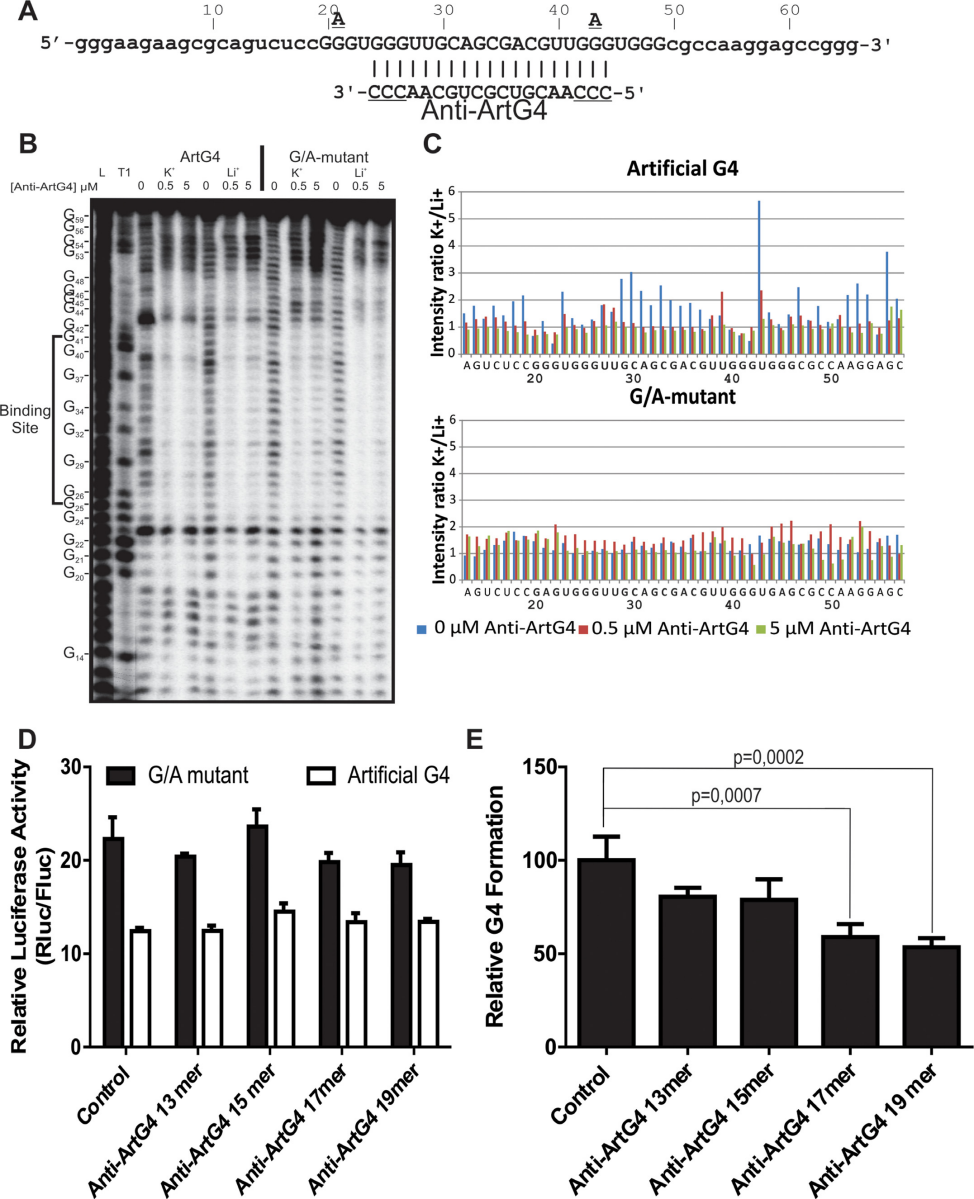
Characterization of the long-loop-2 Artificial G4. (**A**) ArtG4 and Anti-G4 oligonucleotidesequences. G4 nucleotides appear capitals The guanines which were mutated for adenines in the ArtG4 G/A-mutant are shown directly above the ArtG4 residues. Nucleotide numbering is shown directly above the ArtG4 sequence. The Anti-G4 oligonucleotide sequence is located directly beneath the complementary ArtG4 nucleotide stretch; the underlined nucleotides denote those present in the 19-mer but not the shorter ASO. (**B**) Typical autoradiogram of in-line probing of the ArtG4 and G/A-mutant performed in the presence of either 100 mM KCl or LiCl and at three different concentrations of Anti-ArtG4. The two leftmost lanes, designated L and T1, correspond to alkaline hydrolysis and ribonuclease T1 (RNase T1) mappings of ArtG4, respectively. Individual ArtG4 guanosine residues including those encompassed by the Anti-G4 binding site, are indicated along the left edge of the autoradiogram. (**C**) Histograms showing the intensity ratio K^+^/Li^+^, which is an accurate reflection of relative accessibility, for each nucleotide of the ArtG4 and G/A-mutant at three different concentrations of Anti-ArtG4. The average of two independent experiments is shown. (**D**) Luciferase activity of the ArtG4 and G/A-mutant, using different length Anti-artG4 ASO in HEK293. The y axis corresponds to Rluc/Fluc luciferase-activity ratio. (**E**) Relative levels of G4 formation obtained by comparing the ArtG4 and G/A-mutant ratios of luciferase activity obtained using different length Anti-ArtG4 ASO. The G/A mutant/ArtG4 ratio with the control ASO was set at 100, and a ratio equal to 1 was set at 0. Means and standard deviations (s.d.) were calculated from three independent experiments, each conducted in triplicate.

We designed an artificial G4 folding sequence with single-uridine loops 1 and 3, and a 13-nt loop 2 bearing no secondary structure (Figure 2A). To improve resolution for characterization purposes, as well as to better mimic the natural context of an mRNA, ∼15-nt sequences were added on directly in 5′ and 3′ of the G4 motif. These particular ∼15-nt sequences originally flank the native *Fzd2* G4 motif which folds both *in vitro* and *in cellulo* (9) and, thus, should not interfere with folding of the artificial G4 sequence. The 17-mer anti-artificial-G4 ASO (Anti-ArtG4) was designed to simultaneously bind to loop 2 and a pair of guanosine-residue doublets flanking it on each side, in order to efficiently impair G4 formation (Figure 2A). We used 2’O-methyl ribonucleotides, as this chemical modification provides for high-affinity binding (42), *in vivo* stability (43), as well as easy and convenient delivery into mammalian cells (44).

Initially, artificial G4 folding and its modulation by the Anti-ArtG4 ASO were assayed *in vitro* by in-line probing (34,45). Folding of a G4 structure confers added flexibility not only to the loop nucleotides but also to those adjacent to the G tracts, rendering this particular stretch of nucleotides more susceptible to spontaneous non-enzymatic cleavage via in-line nucleophilic attack (9,34). In-line probing requires only trace amounts of RNA (<1 nM), favoring formation of intramolecular G4 topology which is likely more representative of what is found *in vivo*. The artificial G4 was synthesized, purified, 5′-end labeled with ^32^P and then incubated for in-line probing in the presence of either 100-mM LiCl or KCl. Li^+^ is a monovalent cation that does not support G4 formation, whereas K^+^ does (46). Resulting samples were then fractionated by denaturing PAGE. A typical autoradiogram is illustrated in Figure 2B. In the absence of anti-ArtG4 ASO, the presence of K^+^ led to a significant increase of band intensity for loop nucleotides i.e. U_23_, G_29_, C_30_ and U_43_, compared to Li^+^. The four G tracts however were more susceptible to hydrolysis in the presence of Li^+^. The G/A-mutant, created by two G/A substitutions to prevent G4 formation and thus serve as a negative control, displayed no difference in the presence of either cation. The relative intensity of each band was calculated using SAFA software (35). A high ratio of relative band intensity under K^+^ versus Li^+^ conditions (Figure 2C) confirmed that greater accessibility of loop nucleotides occurred when the G4 sequence was in a folded state. Again, no difference was observed in the presence of either K^+^ or Li^+^ for the G/A-mutant. Together, these results confirm that the artificial RNA sequence folded into a G4 structure in the presence of K^+^. This conclusion is further supported by results from CD and thermal denaturation experiments (Supplementary Figure S1A and B and Supplementary Table S3) demonstrating G4 formation solely in the presence of K^+^.

In-line probing was also performed with the anti-ArtG4 ASO at different concentrations (see the Materials and Methods Section for a detailed description). In all cases, the prior addition of anti-ArtG4 ASO abolished G4 folding, as no observable difference was found between band patterns in the presence of K^+^ or Li^+^ (Figure 2B and C). Interestingly, addition of Anti-ArtG4 ASO to pre-folded G4 promoted only limited motif disruption (Supplementary Figure S2). This strongly suggests that while Anti-ArtG4 binding may fail to completely unwind a pre-folded G4, it may still significantly hinder G4 formation by binding to unfolded RNA G4 sequences.

Next, the artificial G4 sequence was inserted into the 5′UTR of a luciferase reporter gene in order to monitor translational modulation *in cellulo*. An artificial G4 G/A-mutant sequence was also inserted, at the same position i.e. 10 nt upstream of the start codon, in a second reporter construct. A random oligonucleotide, used as a negative control, and different length Anti-ArtG4 ASO were transfected separately into HEK293 cells. Transfected cells were grown 24 h, then co-transfected with both luciferase constructs. After an additional 24 h of incubation, cells were lysed and renilla (Rluc) and firefly luciferase (Fluc) activities measured using the dual-luciferase reporter assay. Raw results are displayed in Figure 2D. With the control oligonucleotide, the G/A-mutant showed a 2-fold rise in luciferase expression compared to the regular artificial G4 construct (Figure 2D). This supports the notion that the artificial G4 folds into a stable structure that successfully inhibits translation. Addition of Anti-G4 ASO of different lengths led to distinct phenomena: a slight inhibition of translation of the G/A mutant, and an increase in the artificial G4 sequence expression. One possible explanation is that Anti-ArtG4 duplex formation decreased translation of both the ArtG4 and G/A mutant. However, binding by the ASO also impaired G4 folding, which increased translation of only the ArtG4 mRNA. To quantify the effect that the ASO exerted strictly on G4 formation, and thus take out any other translational effect, an arbitrary reference scale of relative G4 formation was used. This scale compares the ratio of G/A mutant to ArtG4 luciferase activities for each ASO. The result obtained with a random negative-control ASO was arbitrarily set as 100, i.e. 100% of G4 effect observed. A G/A mutant-to-ArtG4 ratio of 1 indicates no difference in luciferase activity i.e. level of translation and, therefore, that no G4 are folded: setting our scale's zero. Since the ASO used should bind with equal affinity to the G/A-mutant and artificial G4 sequences under test conditions, the ratio differences observed with various ASO are logically attributable to altered G4 folding, as opposed to structural alterations or hindrances of ribosomal scanning produced by the ASO. Albeit arbitrary, the proposed scale allowed for quantitative comparisons between ASO, and/or different G4-forming sequences (Figure 2E). The 13-mer Anti-ArtG4 ASO, which binds the central loop but none of the G4 G-tracts, slightly inhibited G4 folding, down to 80%. Conversely, 15-, 17- and 19-mer Anti-ArtG4 ASO bind to the central loop and to 1, 2 or 3 guanines, respectively, of both adjacent G-tracts. This inhibited G4 folding down to 79, 59 and 53%, respectively. Only the results with the 17 and 19 mer were considered statistically significant with a *t*-test. This strongly suggests that as more guanines are bound, less G4 are formed. Therefore, the 19-mer Anti-ArtG4 was used in further experiments.

To ensure that the effects of the Art-G4 and Anti-ArtG4 were exclusively translational and did not affect transcription or mRNA stability, q-PCR experiments were performed. Rluc mRNA levels were identical, irrespective of the ASO used or G4 presence/absence (Supplementary Figure S3A).

We then determined the optimal ASO concentration to inhibit G4 folding *in cellulo*. Concentrations varying between 250 and 1000 ng per well were tested (Supplementary Figure S4). All yielded similar inhibition of G4 folding and were, thus, deemed saturating conditions. A concentration of 750 ng per well was considered as saturating in further experiments, regardless of the targeted G4 or the ASO used. To demonstrate that the observed effects were sequence specific, a mismatched ASO (Anti-mis-ArtG4) was designed. Three residues, at positions originally interacting with loop 2 nucleotides, were modified to create mismatches upon binding of the ASO onto the G4 sequence. Secondary-structure prediction softwares mFold and Pairfold (47,48) were used to prevent the inadvertent altering of either the oligonucleotide's secondary structure or its propensity to form homodimers. Therefore, Anti-mis-ArtG4 should be equally able to bind to potential targets. The ability of the mismatched ASO to modulate folding of the artificial G4 was tested *in cellulo* (Supplementary Figure S5A and B). Luciferase assays revealed that Anti-mis-ArtG4 did not significantly inhibit ArtG4 formation under test conditions, whereas it did modulate folding of an alternative artificial G4 (Alt-ArtG4) bearing three compensatory mutations restoring perfect base-pair complementarity. These results provide important physiological support of the sequence specificity of the observed effects.

### Inhibiting the folding of naturally occurring RNA G4 structures

**Figure 3.**
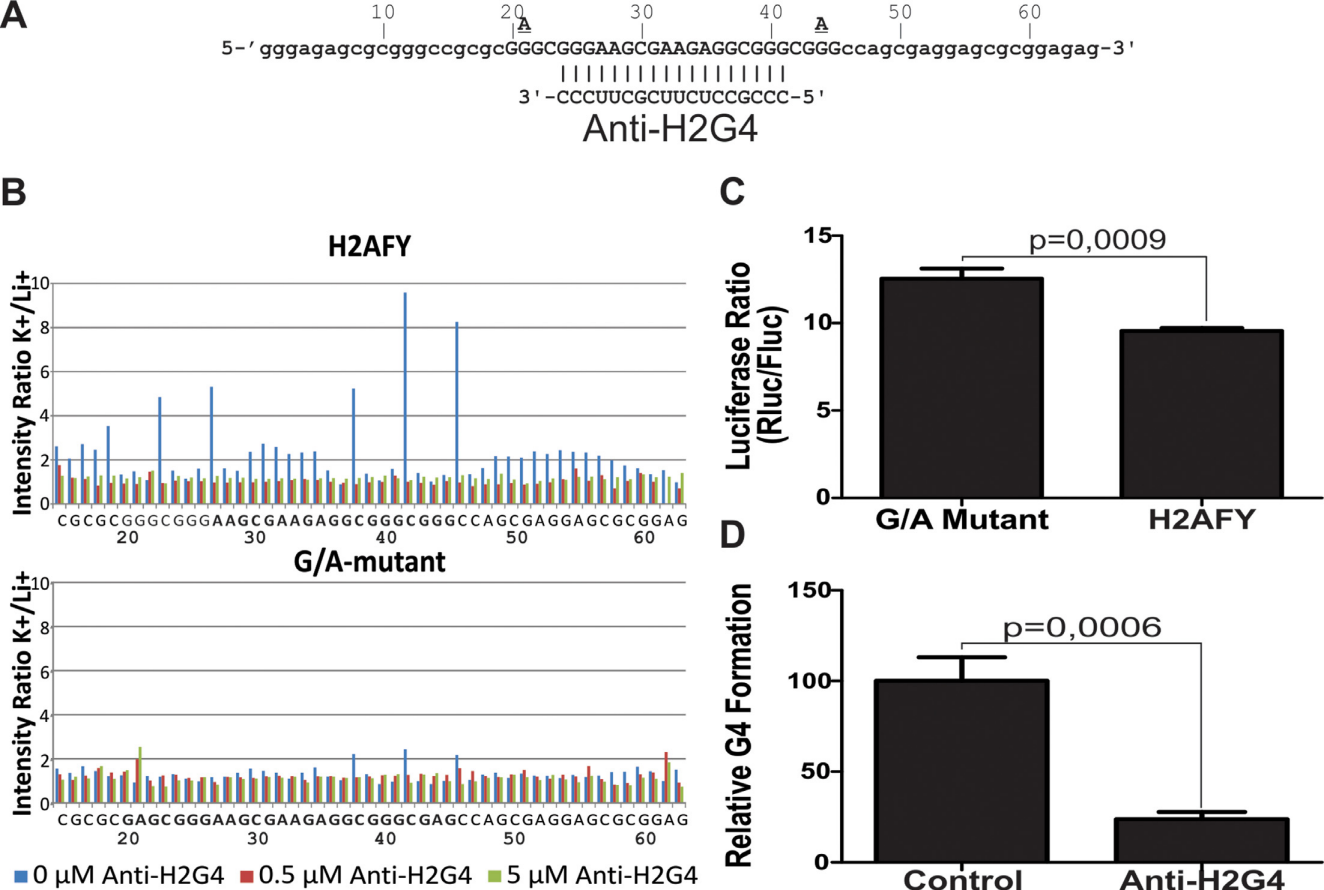
Characterization of naturally-occurring H2AFY G4. (**A**) Sequences of the naturally-occurring H2AFY G4 long loop 2 and the Anti-H2G4 ASO. The H2AFY-G4 nucleotides are capitalized. The guanine residues that were mutated for adenines in the G/A-mutant are shown directly above the H2AFY ASO residues. Nucleotide numbering is shown directly above the H2AFY sequence. The Anti-H2G4 ASO sequence is located directly beneath the complementary H2AFY nucleotide stretch. (**B**) Histograms showing the intensity ratio K^+^/Li^+^, which is an accurate reflection of relative accessibility, for each nucleotide of the H2AFY and G/A-mutant at three different concentrations of Anti-H2G4. The average of two independent experiments is shown. (**C**) Luciferase activity in HEK293 cells for the H2AFY and G/A-mutant. The y-axis corresponds to Rluc/Fluc luciferase activity ratio. (**D**) Relative levels of G4 formation obtained by comparing the H2AFY and G/A mutant ratios of luciferase activity with either a control or Anti-H2G4 ASO. The G/A mutant/H2AFY ratio with the control ASO was set at 100, and a ratio equal to 1 was set at 0. Means and s.d. were calculated from three independent experiments, each conducted in triplicate.

Next, we attempted to demonstrate the feasibility of inhibiting the folding of naturally occurring G4 structures in the 5′UTRs of human genes. Our model target was the H2AFY mRNA. The H2AFY gene codes for two protein isoforms produced from four mRNA isoforms. Importantly, the ratio of both protein isoforms shifts during cellular differentiation (49) and among cancers (31,50,51). Protein isoform 1 is encoded by mRNA isoform 1, whereas Protein isoform 2 by mRNA isoforms 2, 3 and 4. It should be noted that the 5′UTR of mRNA isoforms 1, 3, and 4 all contains a G4 with a 13-nt loop 2 that is targetable by a specific Anti-H2G4 ASO (Figure 3A). The mRNA isoform 2 however contains no G4. Therefore, the totality of mRNAs encoding protein isoform 1 is targetable by the Anti-H2G4 ASO, whereas this is so for only a fraction of mRNAs encoding protein isoform 2. The entire G4 sequence, as well as the ∼20 nt upstream and downstream of the native H2AFY 5′UTR sequence, was transcribed *in vitro* and characterized by in-line probing. Nucleotides located in the loops and near the G tracts of the native sequence became highly susceptible to hydrolysis when transcripts were incubated in the presence of K^+^ compared to Li^+^ (Figure 3B). However, cleavage susceptibility was not affected in a G/A-mutant (Figure 3B), indicating that the H2AFY-derived sequence forms a G4 structure in the presence of K^+^. Results from CD and thermal denaturation experiments further support this conclusion (Supplementary Figure S1C and D and Supplementary Table S3). In-line probing was also performed with an anti-H2G4 ASO specifically targeting loop 2 and both adjacent G tracts (Figure 3A). In the presence of anti-H2G4, susceptibility to hydrolysis of the single-stranded residues fell, indicating inhibition of G4 folding (Figure 3B). Wild type and a G/A-mutant of the full-length H2AFY 5′UTR of the first isoform (NM_001040158.1) were then cloned upstream of a luciferase reporter gene. Both constructs were separately transfected in HEK293 cells, as described above. A small yet statistically significant difference was observed between the constructs (Figure 3C), suggesting that G4 formation decreased levels of translation, albeit not as efficiently as other known G4 (9,10). Folding of this RNA G4 structure seems to have occurred *in vitro* but is limited *in cellulo* by potential competing canonical Watson–Crick secondary structures of the full-length 5′UTR. Such interference has been reported for several other natural G4 (9) and is supported by the results from CD analyses (Supplementary Figure S1C and D and Supplementary Table S3) showing the high stability of the H2AFY G4 sequence under thermal denaturation conditions, even in the presence of Li^+^. Secondary structure prediction data obtained using viennaRNA Package 2.0, with and without the G4-folding module (52), for the H2AYF G4 sequence and 50 nt on each side, further support this hypothesis. Indeed, the predicted canonical Watson–Crick secondary structure comprises the guanines from three out of the four G tracts as part of distinct stem-loops that could possibly interfere and prevent G4 formation (Supplementary Figure S6).

Interestingly, translational rescue was almost complete with the anti-H2G4 ASO which prevented G4 folding. Indeed, only 23% of wild-type H2AFY folded into G4 (Figure 3D). Again, mRNA levels were not significantly different, regardless of the presence/absence of the G4 or ASO, suggesting a translational effect (Supplementary Figure S3B). Importantly, these results confirm that it is feasible to inhibit the folding of a natural human G4 structure using an ASO.

In order to demonstrate the versatility of the ASO approach, we tried to target a different G4, located in the 5′UTR of the Akirin2 mRNA, which encodes a nuclear protein involved in the innate immune response (53). The same assays were performed and results are shown in Supplementary Figure S7. Surprisingly, the addition of the anti-AkG4 ASO did not significantly increase luciferase translation, suggesting that G4 folding was not inhibited (Supplementary Figure S7D). One probable explanation for this is the higher stability of this particular G4 structure (Supplementary Table S3). However, the use of a hybrid DNA/LNA with a phosphothiorate backbone ASO instead of a 2’O-methyl ASO reduced G4 folding to 65%. The higher efficiency of the LNA in G4 targeting is probably due to the better binding to the guanines involved in the G4 folding, as it has been shown that LNA/RNA duplex is more stable than 2’O-Me/RNA duplex (54). The effect of the G4 and ASO occurs at the translational level since mRNA levels remain unchanged (Supplementary Figure S3C). Taken together, these results suggest that optimization of ASO chemistry should help fine-tune and improve inhibition of G4 folding.

### Promoting the folding of RNA G4 structure

**Figure 4.**
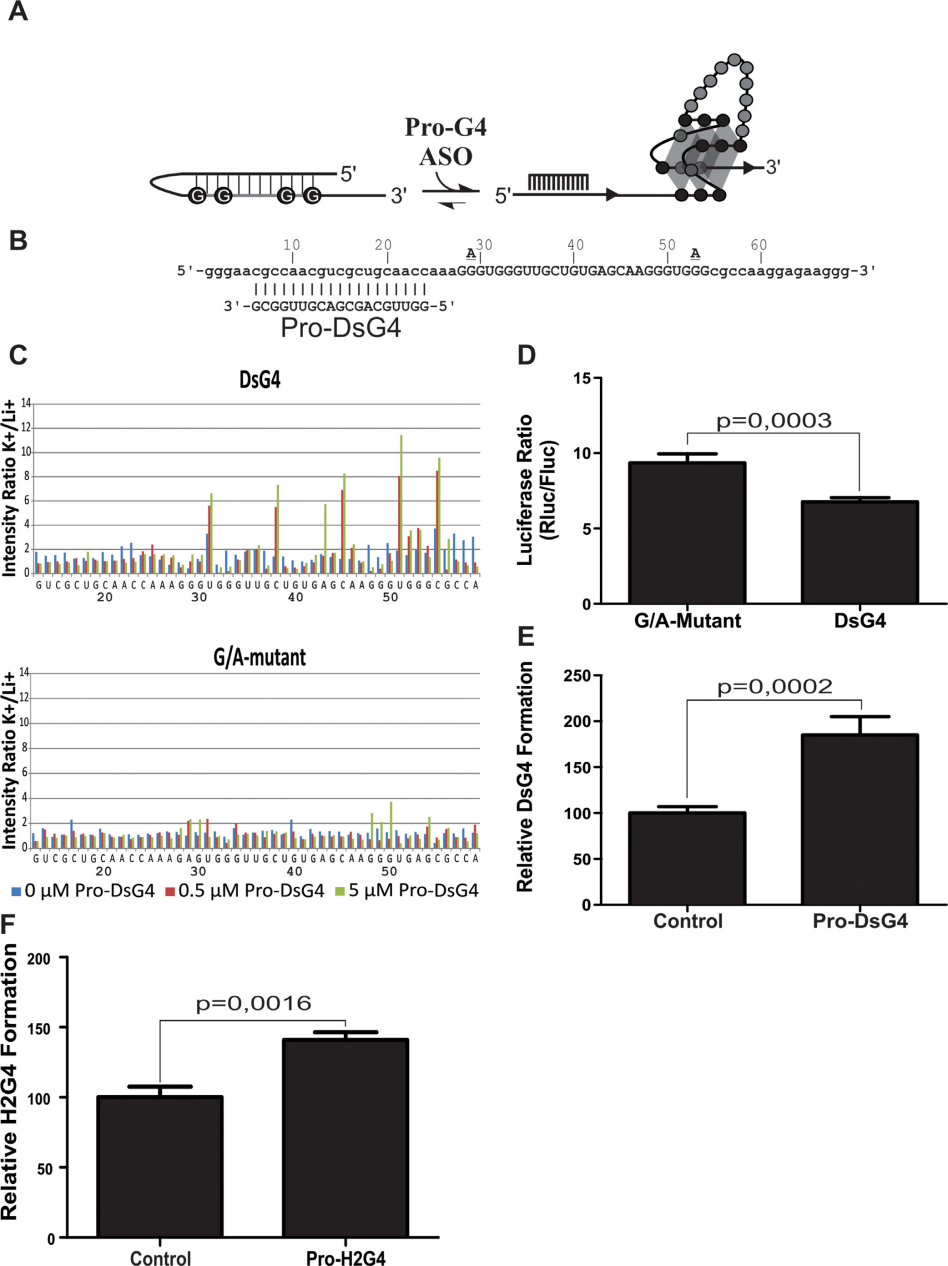
Promoting G4 folding. (**A**) A schematic representationof the ASO-based strategy to promote long-loop-2 G4 folding against the larger nucleotidic context. (**B**) Sequences of the double-stranded G4 (DsG4) and Pro-DsG4 ASO. The G4 nucleotides are capitalized. The guanine residues which were mutated for adenines in the G/A-mutant are shown directly above the respective DsG4 residues. Nucleotide numbering is shown directly above the DsG4 sequence. The Pro-DsG4 oligonucleotide sequence is located directly beneath the complementary DsG4 nucleotide stretch. (**C**) Histograms showing the intensity ratio K+/Li+, which is an accurate reflection of relative accessibility, for each nucleotide of the DsG4 and G/A-mutant at three different concentrations of Pro-DsG4. The average of two independent experiments is shown. (**D**) Luciferase activity in HEK293 cells for the DsG4 and G/A-mutant. (E) and (F) Relative levels of G4 formation obtained by comparing the G/A mutant and DsG4 ratios of luciferase activity with (**E**) a control and the Pro-DsG4 ASO and (**F**) a control and the H2AFY-G4. The G/A mutant/wild type ratio with the control ASO was set at 100, and a ratio equal to 1 was set at 0. Means and s.d. were calculated from four different experiments, each conducted in triplicate.

The H2AFY G4 is a reminder that a G4 structure is a motif that is inherently part of a greater RNA context, the folding of which may be influenced by neighboring sequences. Indeed, surrounding sequences seemed to limit H2AFY G4 formation, as shown above. We wondered if it was possible to design an ASO that would successfully bind a sequence neighboring G tracts that do not fold into a G4 *per se* because they are already engaged in a Watson–Crick-based stable structure. If so, binding of ASO would promote G4 folding and thus enhance translational inhibition (Figure 4A). To test this hypothesis, a novel artificial construct dubbed the ‘double-stranded G4’ (DsG4) was engineered from the initial ArtG4 (Figure 4B). In its initial conformation, the long loop 2 nucleotides and five guanosines that are part of the adjacent G tracts should be included in a stem-loop structure along with the region located upstream of the G4 sequence (Supplementary Figure S8). Binding of the pro-DsG4 ASO, which is complementary to the upstream region of the G4 sequence, was expected to promote release of the G tracts and their folding into a G4 structure. In-line probing experiments were performed on both the DsG4 and a G/A-mutant, with and without the pro-DsG4 ASO (Figure 4C). Interestingly, the sequence seemed to at least partly fold into a G4, even without any ASO, as the nucleotides in the loops and near the G tracts hydrolyzed more readily in the presence of K^+^ compared to Li^+^ (i.e. U_31_, U_51_ and C_55_). Results from CD analyses also showed G4 formation (Supplementary Figure S1E and F). However, the addition of pro-DsG4 led to an important increase in the level of hydrolysis of the same residues as well as nucleotides C_38_ and C_45_ located in loop 2, suggesting that the relative proportion of transcripts adopting the G4 structure was significantly greater in the presence of the ASO. The DsG4 and corresponding G/A-mutant were then cloned upstream of a luciferase reporter gene, and *in cellulo* experiments performed in HEK293 cells as described previously. There was a small yet significant difference in gene expression between the wild type and the G/A-mutant (Figure 4D; 1.3-fold). Upon co-transfection of the pro-DsG4 ASO, this difference markedly increased as more G4 folded (Figure 4E; 185%). Again, no significant changes were observed in mRNA translation levels (Supplementary Figure S3D). Together, these results show that promotion of G4 folding using ASO is also possible *in cellulo*.

Next, the same strategy was applied to the H2AFY G4. We suggested that a secondary structure formed by the 5′UTR would impair G4 folding (see above). Specifically, according to secondary structure prediction, the two first G tracts of the G4 sequence are expected to be involved in a long stem-loop structure (Supplementary Figure S6B). A 19-mer ASO (Supplementary Table S2 for sequence), pro-H2G4, was designed to specifically bind and, thus, disrupt this long stem-loop structure, therefore enabling G4 formation. Co-transfection of pro-H2G4 with the luciferase reporter construct harboring the H2AFY 5′UTR led to a significant increase of G4 folding (Figure 4F, 140%), resulting in a concomitant drop in luciferase activity. As previously observed with other candidates, mRNA levels remained similar (Supplementary Figure S3B). To further demonstrate that results are sequence specific, supplementary control experiments were also performed. Specifically, luciferase assays were conducted with untransfected cells, as well as with cells transfected with a second non-binding control ASO, and with an ASO containing two mismatches (Supplementary Figure S5C). None of these conditions significantly affected relative G4 folding, compared to the first control ASO. This clearly demonstrates that ASO can be used to either inhibit or promote formation of a naturally occurring RNA G4 structure *in cellulo*, resulting in sequence-specific translational activation or repression.

### Targeting endogenous mRNA

**Figure 5.**
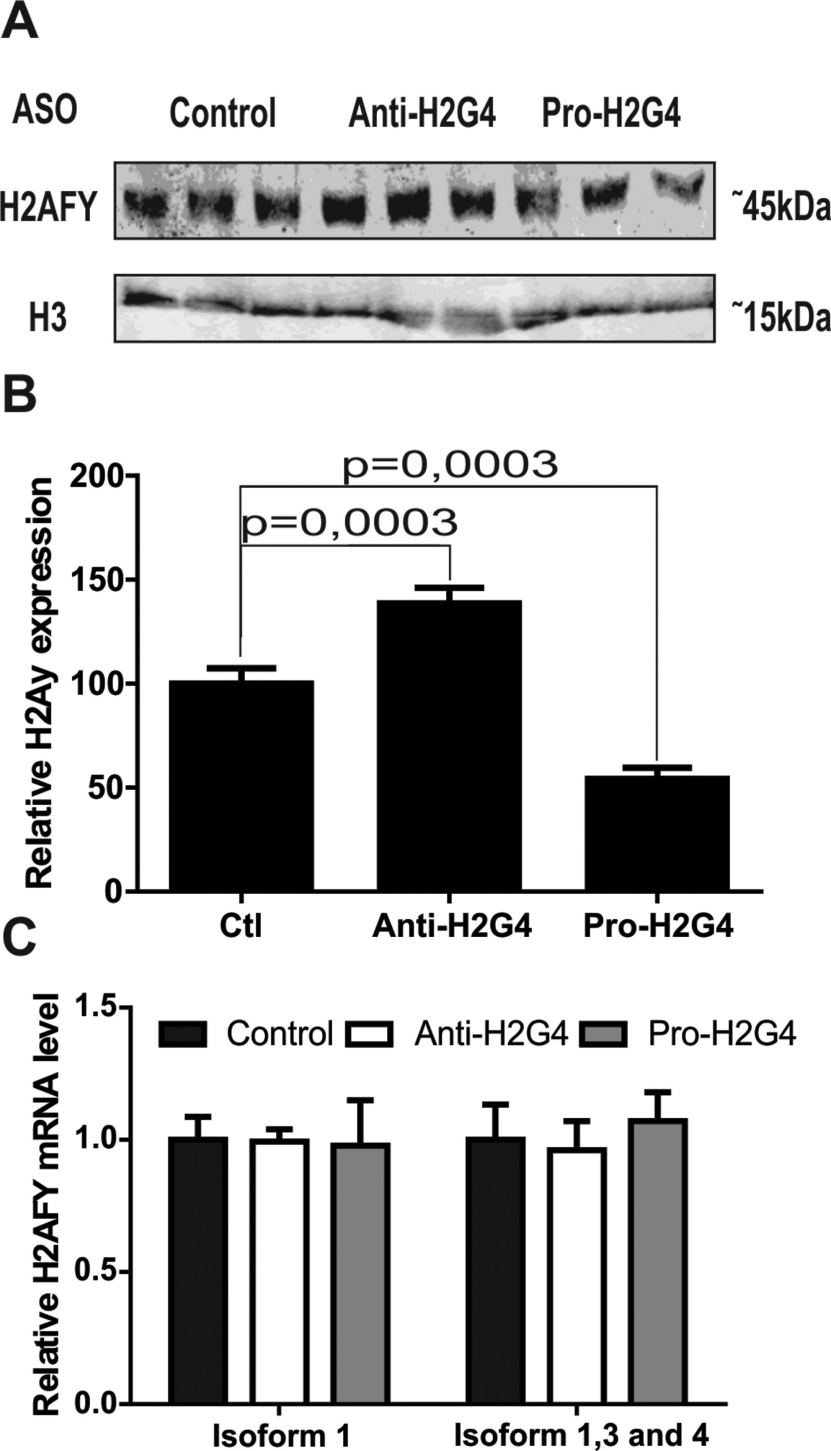
Targeting endogenously expressed H2AFY. (**A**) Typical western blot analysis of Caco-2 cells for the H2AFY protein. Histone 3 (H3) was used as a loading control. Cells were transfected either with the Control, Anti-H2G4 or Pro-H2G4 ASO. (**B**) Quantification of the western blots. Means and SD were calculated from three independent experiments, each conducted in triplicate. (**C**) Relative endogenous mRNA levels for H2AFY isoform 1 (left panel) and isoforms 1, 3 and 4 taken together (right panel) in Caco-2 cells transfected with either the Control (green), Anti-H2G4 (Light green) or Pro-H2G4 (dark green) ASO. Means and SD were calculated from three independent experiments, each conducted in triplicate.

Results from our luciferase assays led us to attempt G4 targeting in endogenously produced H2AFY mRNA. Because H2AFY protein is known to be involved in colorectal cancer (31), we conducted endogenous targeting in Caco-2 cells, a well-recognized cellular model for this particular type of cancer as well as for enterocyte differentiation (55). Caco-2 cells were transfected with either a control, the Anti-H2G4 or the Pro-H2G4 ASO. Western blot and qPCR analyses were then performed to uncover any differences in H2AFY protein expression (Figure 5). The antibody used does not discriminate between the two H2AFY protein isoforms. As expected, transfection of Anti-H2G4 led to higher H2AFY protein levels, whereas the use of Pro-H2G4 led to lower H2AFY protein levels. As observed in the luciferase assays, mRNA isoforms 1, 3 and 4 levels remained unchanged regardless of the transfected ASO, suggesting an effect on translation rather than transcription or mRNA stability. Two distinct pairs of primers were used to detect mRNA isoform 2, unfortunately the concentration appeared to have been below the detection limit of the method (data not shown). These results unambiguously demonstrate that ASO successfully target G4 in endogenous mRNA.

## DISCUSSION

We report the use of ASO to successfully modulate specific RNA G4 formation in human cells. Using ASO, we were able to promote or inhibit translation of reporter genes as well as endogenously expressed mRNAs following modulation of G4 formation. Folding and unfolding of RNA G4 is unmistakably a highly dynamic process (56,57). Several proteins are known to possess DNA or RNA G4 helicase activity (58–60). ASO act as biological tethers, pushing the folding–unfolding equilibrium one way or the other. Here, we observed ∼2-fold variations in translational repression, however RNA G4 are also involved in many other biological mechanisms. Translational repression is certainly the most documented of RNA G4 contributions and we easily monitored translational modulation using luciferase reporter assays. Extending the use of ASO-based strategies to modulate the folding of RNA G4 involved in other fundamental biological cellular mechanisms, such as alternative splicing or polyadenylation, is now within reach. Minute variations in the regulation of such processes are sufficient to lead to significant modifications in gene expression and cellular phenotype (61,62). Accordingly, even small consistent variations in G4 folding through ASO modulation may greatly impact cellular functions. Furthermore, as shown here with the naturally occurring Akirin2 G4, optimization of ASO chemical composition should improve modulation of G4 folding.

ASO-mediated RNA G4 folding is a powerful strategy. Importantly, it also offers an interesting level of specificity, as shown by the successful targeting of a specific and unique G4 sequences *in cellulo*. Relatively short (17–19 nt) ASO were nevertheless long enough to ensure specificity, yet still short enough to avoid off-target effects via non-specific binding. Indeed, we have shown that even a few mismatches greatly reduced targeting efficiency. The actual extent of off-target effects, if any, still requires further investigation but is not expected to be an issue at least for other G4 sequences. Importantly, the observation that modulation of G4 folding occurs in either direction is a good indication that promotion is not simply an artifact of ASO binding, preventing the progress of the ribosome along the mRNA strand.

Another advantage of using ASO is that bidirectional modulation is possible for a single target, as shown here with the H2AFY G4. Interestingly, H2AFY protein isoform 1 is considered a clinically relevant cancer biomarker, including for monitoring of colorectal (31), hepatic (32) and lung (33) cancers. Importantly, the levels of this protein correlate with survival prognoses and progression of metastases. The mechanism by which H2AFY affects cancer development is, however, still poorly understood. Therefore, ASO-mediated modulation of cancer biomarkers, such as H2AFY protein expression, is one more tool that will help move research forward and contribute further insights into their roles and, possibly, provides new clinically relevant therapeutic targets.

ASO offer an interesting alternative to chemical ligands lacking the specificity required for targeting unique G4. Promotion as well as inhibition of G4 folding by ASO is possible. Indeed, ASO-based strategies appear highly specific and provide a powerful set of tools with promising potential for a wide variety of biological applications involving the modulation of G4 motifs.

## SUPPLEMENTARY DATA

Supplementary Data are available at NAR Online.

SUPPLEMENTARY DATA
