# Supplementary material for: Small antisense oligonucleotides against G-quadruplexes: specific mRNA translational switches
Source: Nucleic Acids Res. 2014 Dec 15;43(1):595–606. doi: 10.1093/nar/gku1311 (PMC4288198; doi:10.1093/nar/gku1311)
Supplement: SUPPLEMENTARY DATA [file supp_43_1_595__index.html]

Small antisense oligonucleotides against G-quadruplexes: specific mRNA translational switches — SUPPLEMENTARY DATA 

# Small antisense oligonucleotides against G-quadruplexes: specific mRNA translational switches

## SUPPLEMENTARY DATA

**Files in this Data Supplement:**

- SUPPLEMENTARY DATA
