# Supplementary material for: Small antisense oligonucleotides against G-quadruplexes: specific mRNA translational switches
Source: Nucleic Acids Res. 2014 Dec 15;43(1):595–606. doi: 10.1093/nar/gku1311 (PMC4288198; doi:10.1093/nar/gku1311)
Supplement: SUPPLEMENTARY DATA [file supp_gku1311_Supplementary-Informations.docx]

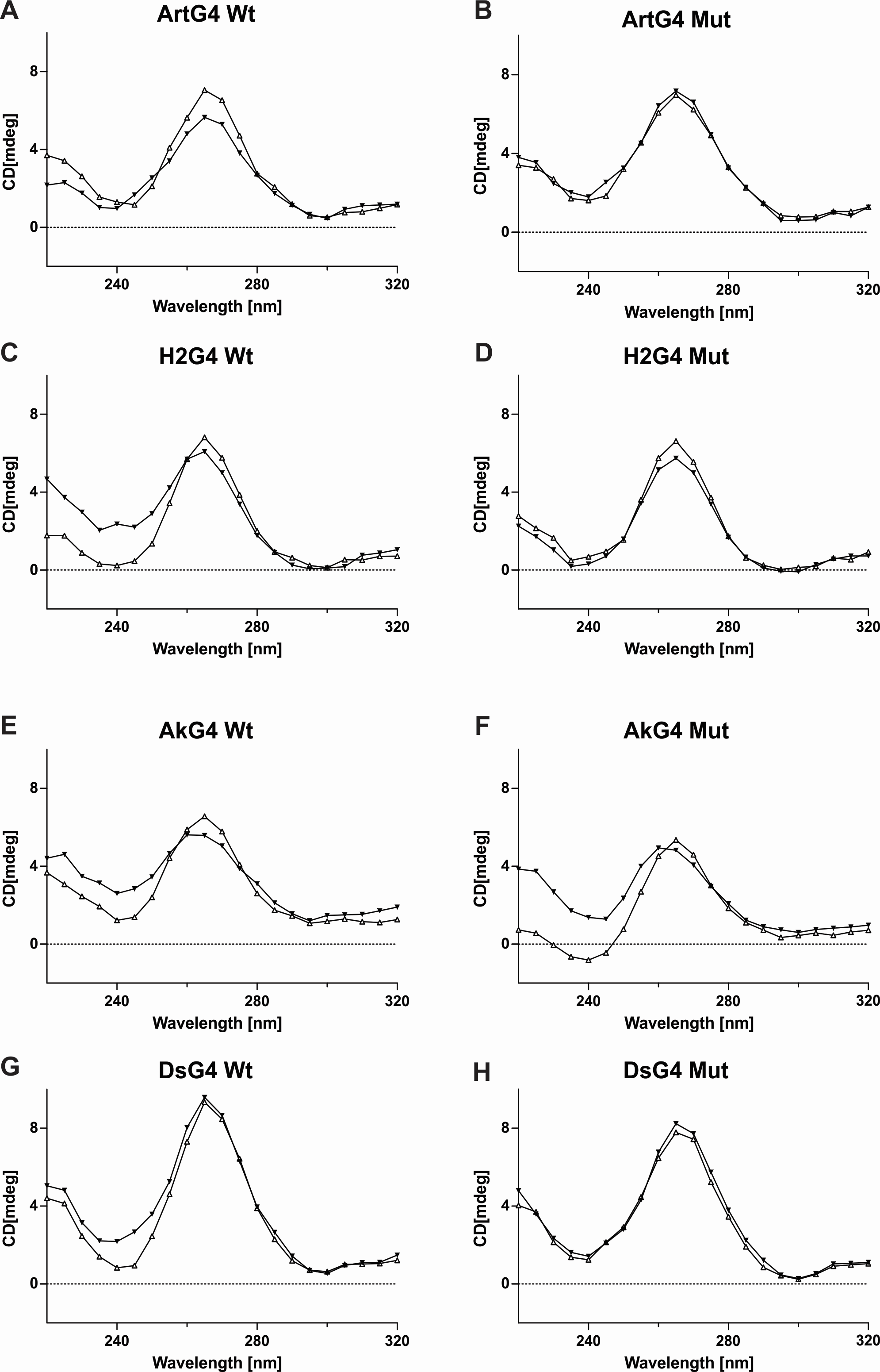


**Supplementary Figure 1: Comparative circular-dichroism analysis of G-Quadruplexes.** Circular-dichroism spectra for the ArtG4 (a), ArtG4 G/A mutant (b), H2AFY G4 (c), H2AFY G/A mutant (d), Akirin2 G4 (e), and Akirin2 G4 G/A mutant (f),DsG4 (g), DsG4 G/A mutant (h), using 4 mM of respective sequence in the presence of either 100mM LiCl (black triangles), or 100 mM KCl (white triangles).


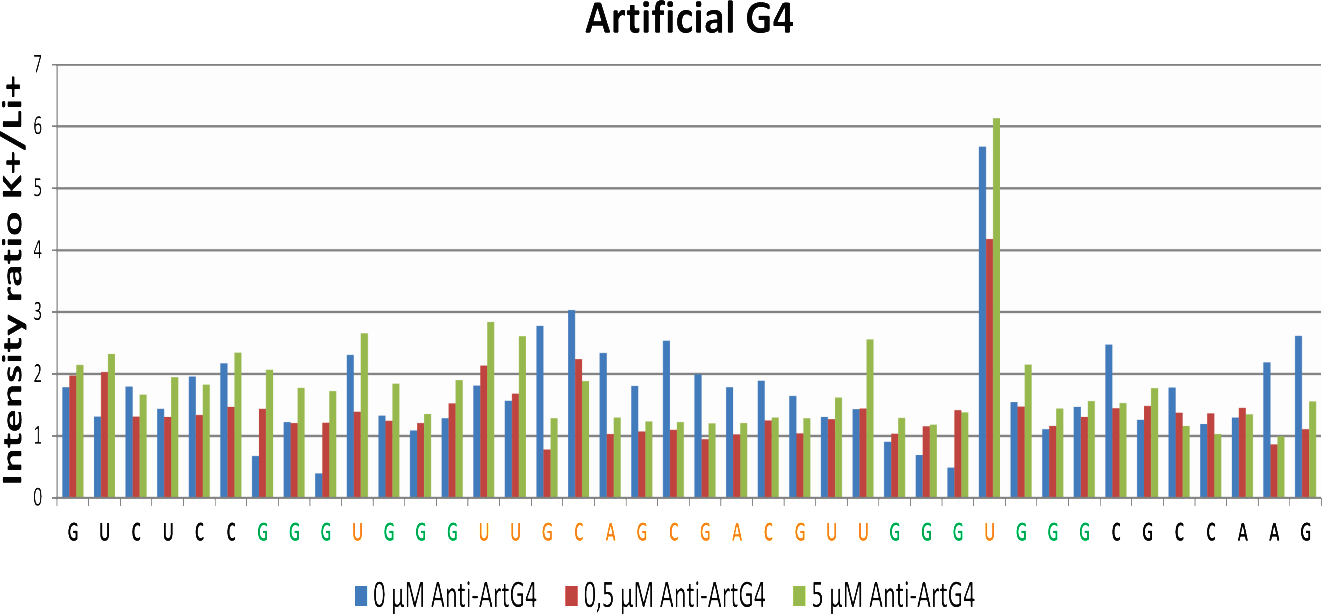


**Supplementary Figure 2:** **Anti-ArtG4 cannot disrupt pre-folded ArtG4.** Histograms showing the relative intensity ratio K^+^/Li^+^, which is an accurate reflection of relative accessibility, for each nucleotide of the pre-folded ArtG4 and G/A-mutant, with different concentrations of Anti-ArtG4 ASO added after G4 folding.


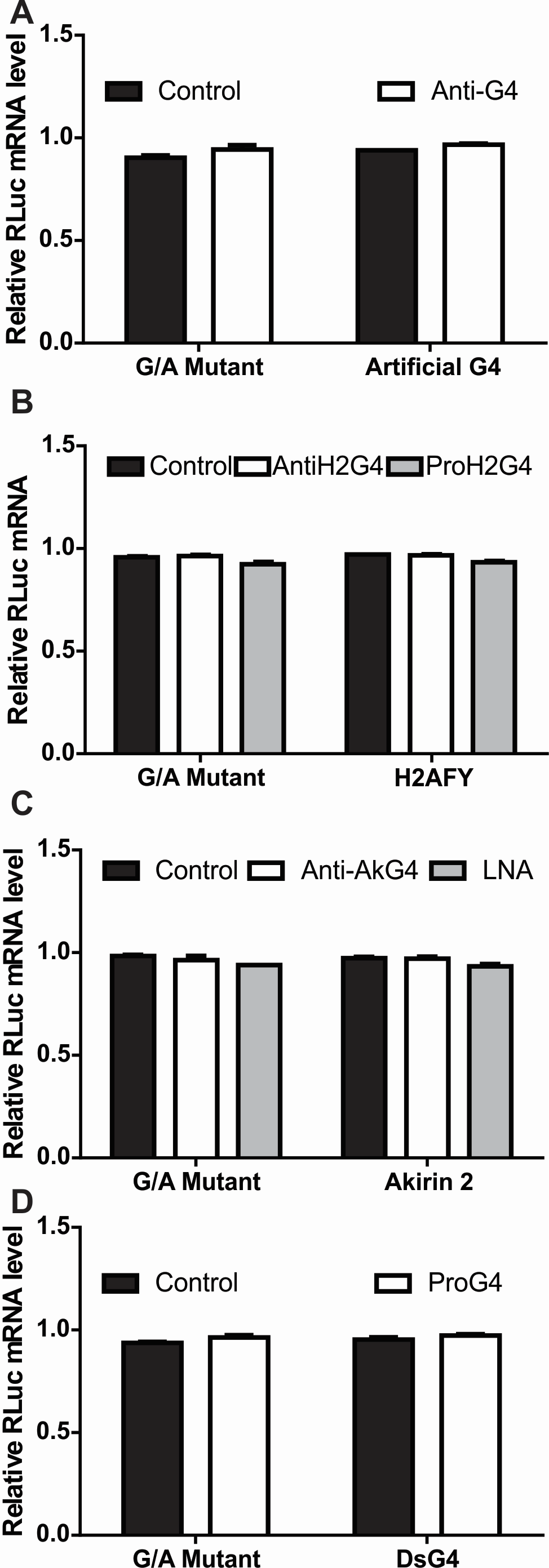


**Supplementary Figure 3: Modulation of G4 folding does not affect mRNA levels.** Rluc mRNA levels were assayed with q-PCR and normalized using Fluc mRNA levels. Wild-type and G/A-mutant Rluc mRNA levels were compared for the ArtG4 (a), H2AFY G4 (b), Akirin2 G4 (c), and DsG4 (d), using control and other ASO (see the color legends of each panel). Means and s.d. were calculated from three independent experiments, each conducted in triplicate.

**
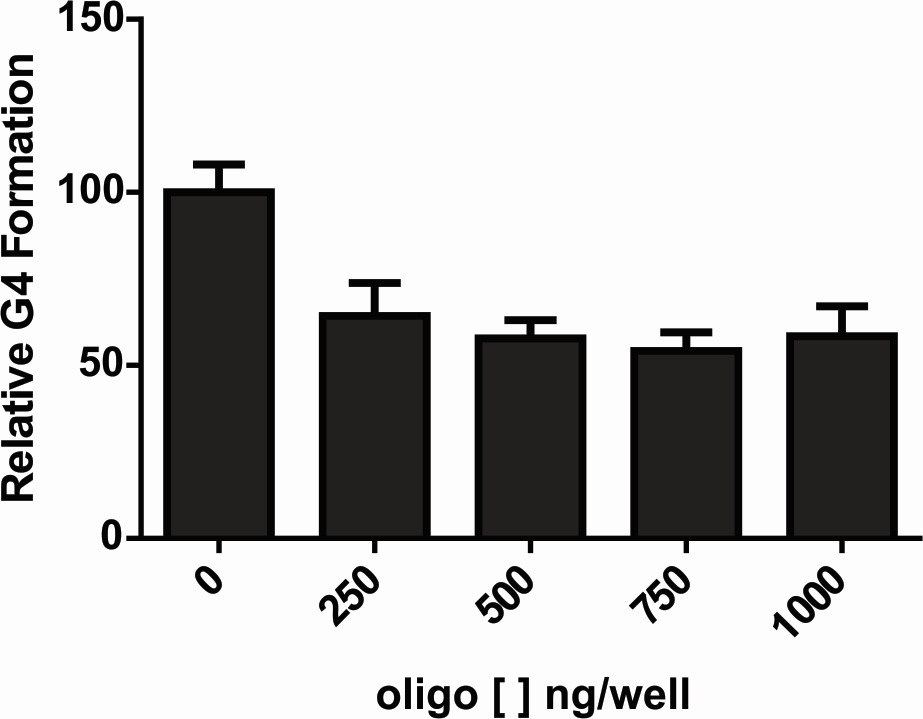
**

**Supplementary Figure 4: Dose response of the ArtG4 to Anti-ArtG4 ASO.** Relative G4 formation obtained by comparing ArtG4 G/A mutant/wild-type ArtG4 luciferase activity ratio, at four different concentrations of Anti-ArtG4 ASO. Means and s.d. were calculated from three independent experiments, each conducted in triplicate.

**
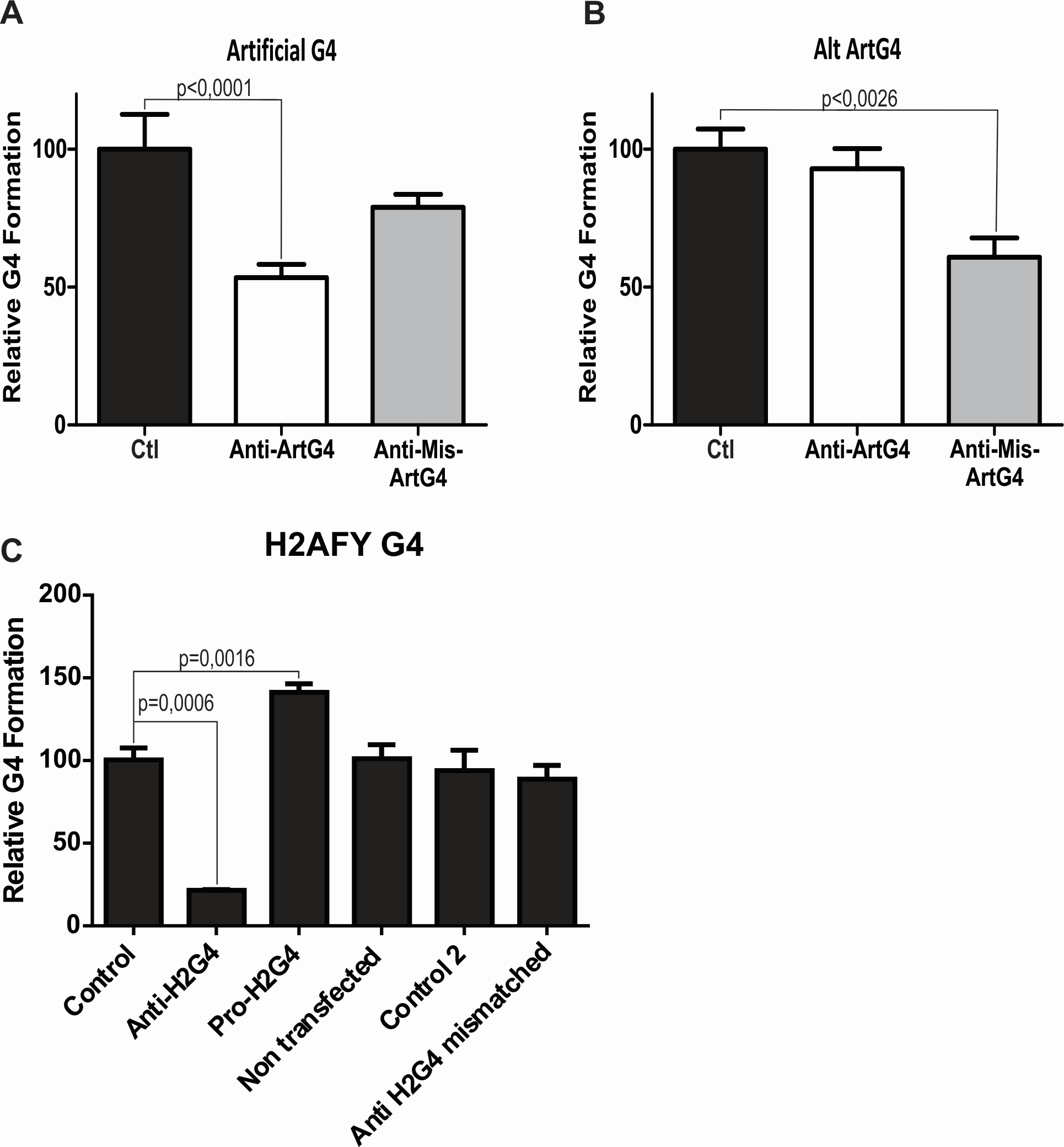
**

**Supplementary Figure 5: Modulation of G4 folding is sequence specific.** Relative G4 formation obtained by comparing the G/A Mutant/Wild-type G4 luciferase activity for the ArtG4 (A) and the Alt ArtG4 (B) using control, Anti-ArtG4 or Anti-MisG4 ASO. (C) Relative G4 formation of the H2AFY G4 using Control, Anti-H2G4, Pro-H2G4 ASO (as shown in Figures 3 and 4), non-transfected cells, a second non-binding Control (Control 2) namely the Pro-DsG4 used to target the DsG4 construct, and mismatched Anti-H2G4 ASO. In all cases, the ratio obtained with the control ASO was set at 100 and a ratio equal to 1 was set at 0. Means and s.d. were calculated from four independent experiments, each conducted in triplicate.


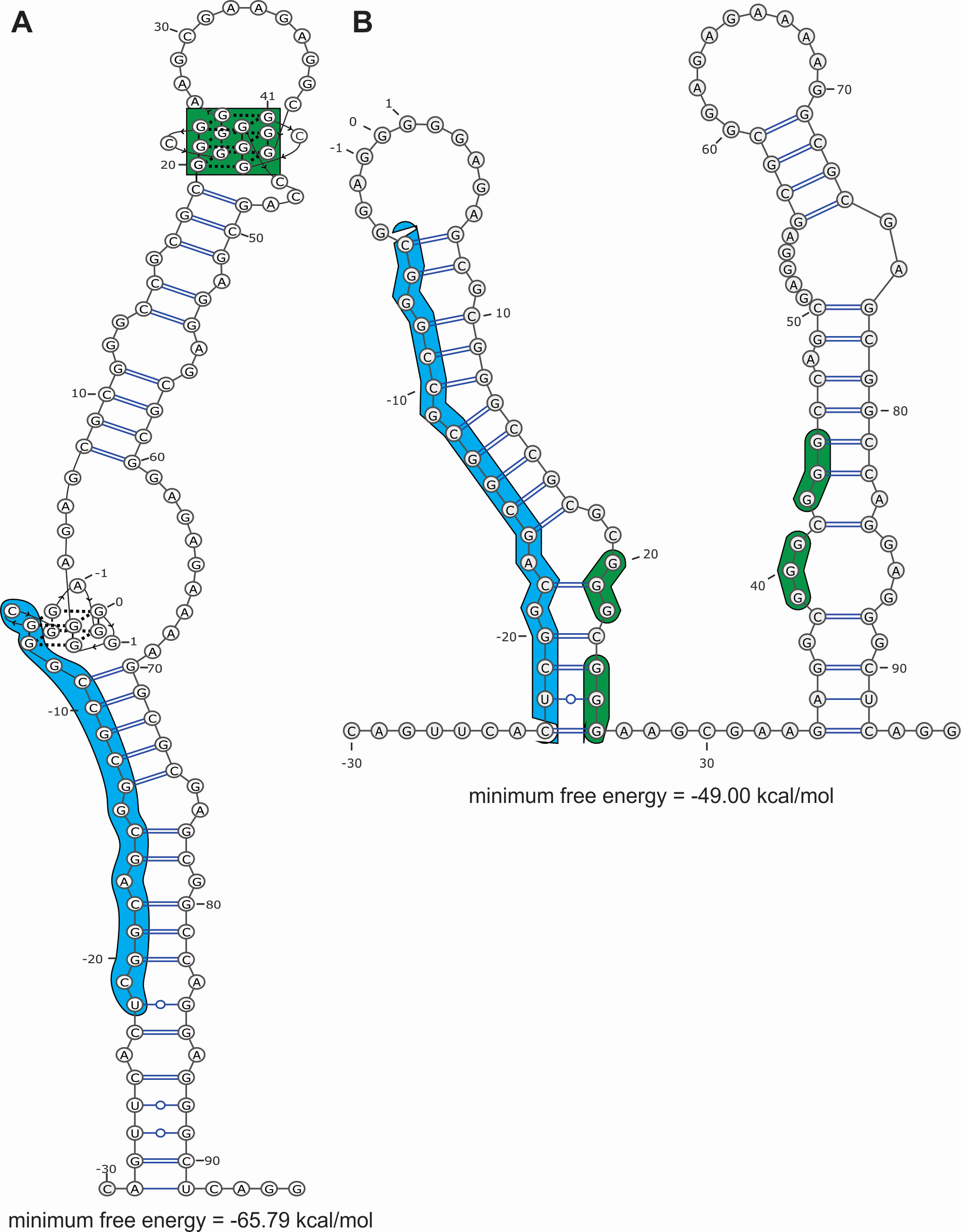


**Supplementary Figure 6: Structural context of the H2AFY G4.** Predicted secondary structures of the 26-nt H2AFY G4 stretch including the 50-nt sequences directly upstream and downstream of the G4, obtained using RNAfold, with or without the G4-predicting module. Nucleotide numbering is based on that of the sequence that was transcribed for in-line probing and corresponds to that shown in Figure 3. The guanine residues that are part of the G4 structure are highlighted in green. The residues bound by Pro-H2G4 ASO are highlighted in blue.

**
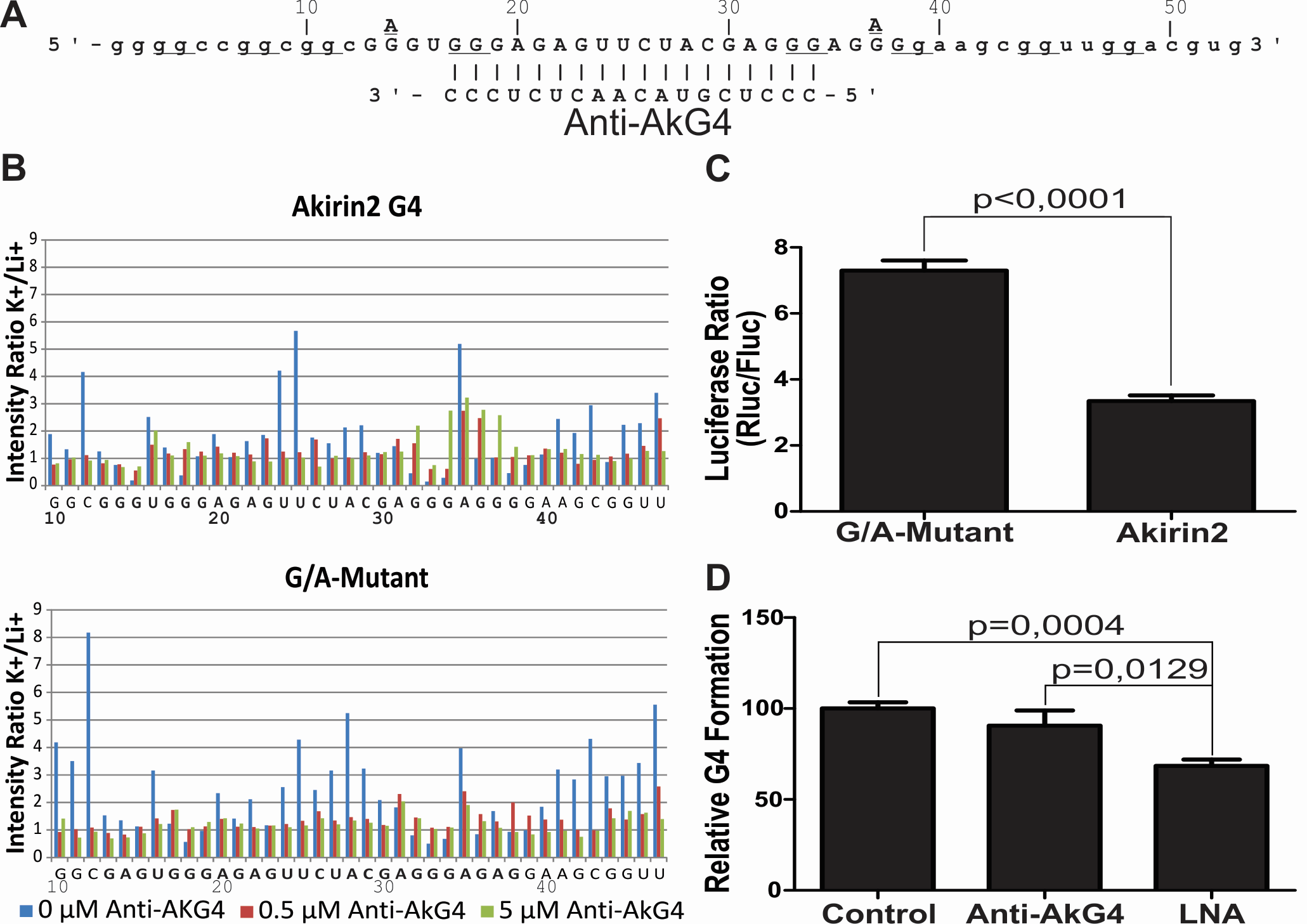
**

**Supplementary Figure 7: Characterization of the naturally-occurring AkG4**. (a) Sequences of the naturally-occurring long-loop 2 AkG4 and Anti-AkG4 ASO. The G4 residues are capitalized in. The guanines residues which were mutated for adenines in the G/A-mutant, as well as the nucleotide numbers are shown directly above the respective AkG4 residues. The Anti-AkG4 ASO oligonucleotide sequence is located directly beneath the complementary AkG4 nucleotide stretch. (b) Histograms showing the intensity ratio K^+^/Li^+^, which is an accurate reflection of relative accessibility, for each nucleotide of the wild-type and G/A-mutant AkG4 using three different concentrations of Anti-AkG4 ASO. The average of two independent experiments is shown. The AkG4 G/A mutant seems to form a double-quartet G4 using the guanines residues underlined in (a). (c) Luciferase activity in HEK293 cells for the AkG4 and G/A mutant. (d) Relative levels of G4 formation obtained by comparing the G/A mutant/AkG4 ratio of luciferase activity using different ASO. The G/A mutant/AkG4 ratio with the control ASO was set at 100, and a ratio equal to one was set at 0. Means and s.d. were calculated from three independent experiments, each conducted in triplicate.


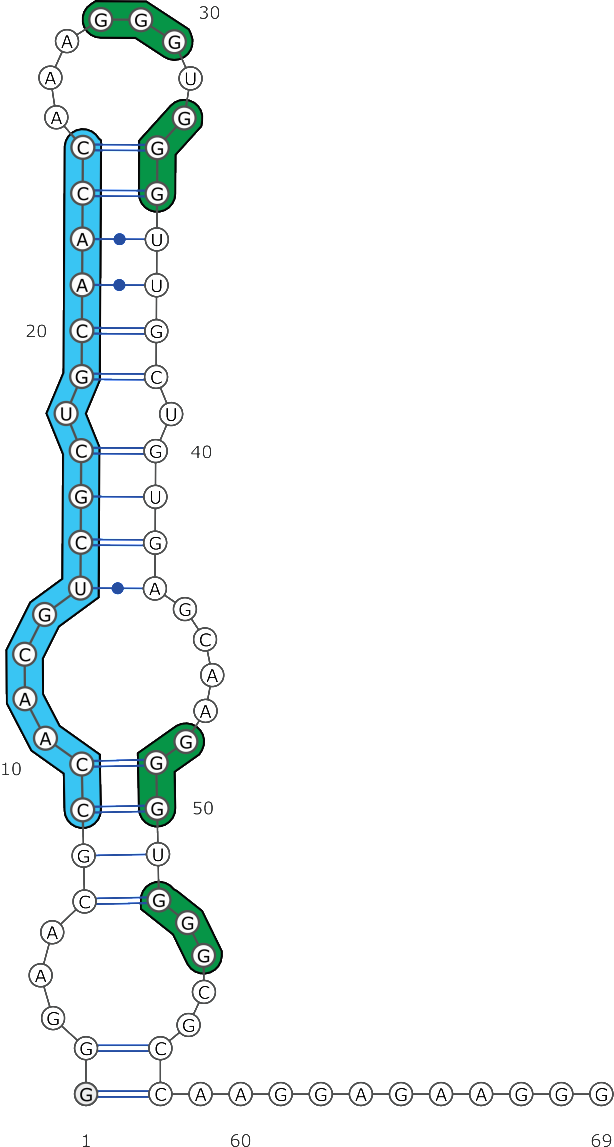


**Supplementary Figure 8: DsG4 structure** Prediction of the secondary structure of the DsG4, obtained using RNAfold. The guanine residues that are part of the G4 structure are highlighted in green. The residues bound by the Pro-DsG4 ASO are highlighted in blue.

**Supplementary Table 1: G4 ASO**

Names and nucleotide sequences of ASO required for synthesis (syn) of the study G4 (wt) and their G/A-mutant (mut) counterparts, as well as for construction of the study 5’UTR (5UTR), and modulation of G4 folding (Anti or Pro). For the LNA version of Anti-AkG4, the LNA residues are underlined.

| **G4** | **Name** | **5’- Sequence -3’** |
| --- | --- | --- |
| *ArtG4* | Syn-wt | CCCGGCTCCTTGGCGCCCACCCAACGTCGCTGCAACCCACCCGGAGACTGCGCTTCTTCCCtatagtgagtcgtatta |
|  | Syn mut | CCCGGCTCCTTGGCGCtCACCCAACGTCGCTGCAACCCACtCGGAGACTGCGCTTCTTCCCtatagtgagtcgtatta |
|  | 5UTR-forward wt | ctagcgggaagaagcgcagtctccGGGTGGGTTGCAGCGACGTTGGGTGGGcgccaaggagccgggg |
|  | 5UTR-reverse wt | CTAGCTCCCGGCTCCTTGGCGCCCTCCCUUCGTCGCTGCTTCCCTCCCGGTGTCTGCGCTTCTTCCCG |
|  | 5UTR-forward mut | ctagcgggaagaagcgcagtctccGAGTGGGTTGCAGCGACGTTGGGTGAGcgccaaggagccgggg |
|  | 5UTR-reverse  Mut | CTAGCCCCGGCTCCTTGGCGCTCTCCCTTCGTCGCTGCTTCCCTCTCGGTGTCTGCGCTTCTTCCCG |
|  | Anti-ArtG4  (17mer) | CCAACGUCGCUGCAACC |
|  | Anti-ArtG4  (19mer) | CCCAACGUCGCUGCAACCC |
| *Alt ArtG4* | Syn-wt | CCCGGCTCCTTGGCGCCCACCCACGGTCGCTTCAACCCACCCGGAGACTGCGCTTCTTCCCtatagtgagtcgtatta |
|  | Syn mut | CCCGGCTCCTTGGCGCTCACCCACGGTCGCTTCAACCCACTCGGAGACTGCGCTTCTTCCCtatagtgagtcgtatta |
|  | 5UTR-forward wt | CTAGCGGGAAGAAGCGCAGTCTCCGGGTGGGTTGaAGCGACcgTGGGTGGGCGCCAAGGAGCCGGGG |
|  | 5UTR-reverse wt | CTAGCCCCGGCTCCTTGGCGCCCACCCACGGTCGCTTCAACCCACCCGGAGACTGCGCTTCTTCCCG |
|  | 5UTR-forward mut | CTAGCGGGAAGAAGCGCAGTCTCCGaGTGGGTTGaAGCGACcgTGGGTGaGCGCCAAGGAGCCGGGG |
|  | 5UTR-reverse  Mut | CTAGCCCCGGCTCCTTGGCGCtCACCCACGGTCGCTTCAACCCACtCGGAGACTGCGCTTCTTCCCG |
|  | Anti-misG4 | CCCACGGUCGCUUCAACCC |
| *H2AFY G4* | Syn-wt | CTCTCCGCGCTCCTCGCTGGCCCGCCCGCCTCTTCGCTTCCCGCCCGCGCGGCCCGCGCTCTCCCtatagtgagtcgtatta |
|  | Syn-mut | CTCTCCGCGCTCCTCGCTGGCtCGCCCGCCTCTTCGCTTCCCGCtCGCGCGGCCCGCGCTCTCCCtatagtgagtcgtatta |
|  | 5UTR-1 | ACTGGTTCCAGTTCACTCGGCAGCGGCGCCGGGCGGAGGGGGAGAGCGCGGGCCGCGC |
|  | 5UTR-2 wt | CGCCTTTTCTCTCCGCGCTCCTCGCTGGCCCGCCCGCCTCTTCGCTTCCCGCCCGCGCGGCCCGCGCTCTCC |
|  | 5UTR-2 mut | CGCCTTTTCTCTCCGCGCTCCTCGCTGGCtCGCCCGCCTCTTCGCTTCCCGCtCGCGCGGCCCGCGCTCTCC |
|  | 5UTR-3 | GGAGCGCGGAGAGAAAAGGCGCGAGCGGCCAGGAGGGCTCAGGCCGAGACACCTTGCAGCTGCC |
|  | 5UTR-4 | GGCGGTGGCCCTGGAGGCGGATCAGTGAGCACAGCGGCGGCTCGGTGGCGGCGGCGGCAGCTGCAAGGTGTC |
|  | 5UTR-Forward | TCAGTCAgctagcACTGGTTCCAGTTCACTCGG |
|  | 5UTR-Reverse | TCAGTCAgctagcGGCGGTGGCCCTGGAGGCG |
|  | Anti-H2G4 | CCCGCCUCUUCGCUUCCC |
|  | Pro-H2G4 | GCCCGGCGCCGCUGCCGAG |
|  | Anti-misH2G4 | CCCGCCUCUUCCGUUCCC |
| *DsG4* | Syn-wt | CCCTTCTCCTTGGCGCCCACCCTTGCTCACAGCAACCCACCCTTTGGTTGCAGCGACGTTGGCGTTCCCtatagtgagtcgtatta |
|  | Syn mut | CCCTTCTCCTTGGCGCtCACCCTTGCTCACAGCAACCCACtCTTTGGTTGCAGCGACGTTGGCGTTCCCtatagtgagtcgtatta |
|  | 5UTR-forward wt | CTAGCgggaacgccaacgtcgctgcaaccaaaGGGTGGGTTGCTGTGAGCAAGGGTGGGcgccaaggagaagggG |
|  | 5UTR-reverse wt | CTAGCCCCTTCTCCTTGGCGCCCACCCTTGCTCACAGCAACCCACCCTTTGGTTGCAGCGACGTTGGCGTTCCCg |
|  | 5UTR-forward mut | CTAGCgggaacgccaacgtcgctgcaaccaaaGaGTGGGTTGCTGTGAGCAAGGGTGaGcgccaaggagaagggG |
|  | 5UTR-reverse  Mut | CTAGCCCCTTCTCCTTGGCGCTCACCCTTGCTCACAGCAACCCACTCTTTGGTTGCAGCGACGTTGGCGTTCCCG |
|  | Pro-DsG4 | GGUUGCAGCGACGUUGGCG |
| *Akirin2* | Syn-wt | CACGTCCAACCGCTTCCCCTCCCTCGTAGAACTCTCCCACCCGCCGCCGGCCCCtatagtgagtcgtatta |
|  | Syn-mut | CACGTCCAACCGCTTCCtCTCCCTCGTAGAACTCTCCCACtCGCCGCCGGCCCCtatagtgagtcgtatta |
|  | 5UTR-Forward mut | AGTCAgctagcTATTGACGCCATATTGGGGCCGGCGGCGtGTGGGAGAGTTCTACGAGGGAGtGGAAGCGGTTGGACGTGTTCGCTTGGG |
|  | 5UTR-Reverse | TCAGTCAgctagcGGCCGGGGGCAGCTGAGGC |
|  | Anti-AkG4 | CCCUCGUAGAACUCUCCC |
| *All* | Control | AUUUUCUCCUCGCAAUUUA |

**Supplementary Table 2: Primers used for q-PCR**

| **Fwd Name** | **Rev Name** | **Fwd Seq** | **Rev Seq** |
| --- | --- | --- | --- |
| fLuc.q.F2 | fLuc.q.R2 | GTGGGCAAGGTGGTGCCATT | AATCATAGGGCCGCGCACAC |
| rLuc.q.F2 | rLuc.q.R2 | AAGGGCCTCCACTTCAGCCA | TTCTTCAGCACGCGCTCCAC |
|  |  |  |  |
| H2AFY.iso1.q.F2 | H2AFY.iso1.q.R2 | GCCGACAGCACAACCGAGGG | GGGTGAACGACAGCATCACTGT |
| H2AFY.iso1_3_4.q.F2 | H2AFY.iso1_3_4.q.R2 | GGCCGAGACACCTTGCAGCT | TGGACTTCTTCTTCCCACCGCG |
| H2AFY.iso2.q.F2 | H2AFY.iso1_3_4.q.R2 | GGCAGGTTCCAGACTTCGGGG | TGGACTTCTTCTTCCCACCGCG |
| H2AFY.iso2.q.F3 | H2AFY.iso2.q.R4 | GGAGGATTAACTGAAGGACCCCAGG | TGATGTACCGCAGCATCCGC |
|  |  |  |  |
| MRPL19_G_3_f | MRPL19_G_3_r | AAGGAGAAAAGTACTCCACATTCCAGAG | TGGGTCAGCTGTAGTAACACGA |
| SDHA_G_f | SDHA_G_r | TGTTGATGGGAACAAGAGGGCA | GCCTACCACCACTGCATCAAAT |
| YWHAZ_G_f | YWHAZ_G_r | TCCCCAATGCTTCACAAGCAGA | TCTTGTCATCACCAGCGGCAA |

**Supplementary Table 3: Thermal denaturation analysis**

| **G-quadruplexes** | | **Li^+^** | **K^+^** | **∆*T*_m_** |
| --- | --- | --- | --- | --- |
| *ArtG4* | Wt | 68.2 ± 1.9 | 82.3 ± 0.9 | 14.1 |
|  | Mut | 62.4 ± 1.5 | 64.1 ± 0.1 | 1.7 |
| *H2AFY G4* | Wt | 85.8 ± 1.8 | >90 | >4.2 |
|  | Mut | 80.6 ± 0.1 | 76.8 ± 0.3 | -3.8 |
| *DsG4* | Wt | 59.7 ± 1.1 | 84.7 ± 1.5 | 25.0 |
|  | Mut | 71.2 ± 0.7 | 69.8 ± 0.4 | -1.4 |
| *Akirin2 G4* | Wt | 54.7 ± 2.0 | >90 | >35.3 |
|  | Mut | 56.3 ± 2.7 | 64.8 ± 0.3 | 8.48 |

Values are means calculated from 2 independent experiments and ± correspond to s.d.
